# Gender differences in the association of individual and contextual socioeconomic status with hypertension in 230 Latin American cities from the SALURBAL study: a multilevel analysis

**DOI:** 10.1186/s12889-023-16480-3

**Published:** 2023-08-11

**Authors:** Débora Moraes Coelho, Amanda Cristina de Souza Andrade, Uriel Moreira Silva, Mariana Lazo, S. Claire Slesinski, Alex Quistberg, Ana V. Diez-Roux, Amélia Augusta de Lima Friche, Waleska Teixeira Caiaffa

**Affiliations:** 1 Faculty of Medicine, Federal University of Minas Gerais, Avenida Alfredo Balena 190, Belo Horizonte, 30130-100 Brazil; 2 Belo Horizonte Observatory for Urban Health, Avenida Alfredo Balena 190, Belo Horizonte, 30130-100 Brazil; 3 Institute of Public Health, Federal University of Mato Grosso, Avenida Fernando Corrêa 2367, Cuiabá, 78060-900 Brazil; 4 Dornsife School of Public Health, Drexel University, 3215 Market Street, Philadelphia, PA 19104 USA

**Keywords:** Urban health, Hypertension, Education, Socioeconomic status, Latin America, Multilevel analysis

## Abstract

**Background:**

Despite global interest in gender disparities and social determinants of hypertension, research in urban areas and regions with a high prevalence of hypertension, such as Latin America, is very limited.

The objective of this study was to examine associations of individual- and area-level socioeconomic status with hypertension in adults living in 230 cities in eight Latin America countries.

**Methods:**

In this cross-sectional study, we used harmonized data from 109,184 adults (aged 18–97 years) from the SALURBAL (Salud Urbana en America Latina/Urban Health in Latin America) project. Hypertension was assessed by self-report. Individual-, sub-city- and city-level education were used as proxies of socioeconomic status. All models were stratified by gender.

**Results:**

Higher individual-level education was associated with lower odds of hypertension among women (university education or higher versus lower than primary: odds ratio [OR] = 0.67, 95% confidence interval [CI] = 0.61–0.74) but higher odds among men (OR = 1.65; 95%CI 1.47–1.86), although in men an inverse association emerged when measured blood pressure was used (OR = 0.86; 95%CI 0.76–0.97). For both genders, living in sub-city areas with higher educational achievement was associated with higher odds of hypertension (OR per standard deviation [SD] = 1.07, 95%CI = 1.02–1.12; OR = 1.11 per SD, 95%CI = 1.05–1.18, for women and men, respectively). The association of city-level education with hypertension varied across countries. In Peru, there was an inverse association (higher city level education was associated with lower odds of hypertension) in women and men, but in other countries no association was observed. In addition, the inverse association of individual-level education with hypertension became stronger (in women) or emerged (in men) as city or sub-city education increased.

**Conclusion:**

The social patterning of hypertension differs by gender and by the level of analysis highlighting the importance of context- and gender-sensitive approaches and policies to reduce the prevalence of hypertension in Latin America.

**Supplementary Information:**

The online version contains supplementary material available at 10.1186/s12889-023-16480-3.

## Background

Hypertension, a major contributor to cardiovascular disease [1], is a major global health challenge and disproportionately affects populations in low- and middle-income countries (LMICs) [2]. Recently, studies have gone beyond traditional behavioral risk factors and have shown that the individual’s socioeconomic conditions and the contextual characteristics of the places where people live are related to hypertension prevalence [3–5]. Although socioeconomic conditions have been identified as important factors in increasing the burden of hypertension in LMICs [1], evidence is limited and results are not always consistent in the context of Latin America [6, 7].

In high-income countries, studies have generally reported an inverse association of hypertension with socioeconomic status (SES) at the individual level [6] and at the area level [8, 9]. In contrast, evidence on the association of individual-level and area-level SES with hypertension in Latin America remains comparatively scarce and conflicting. At the individual level, some studies reported an inverse association of SES with hypertension [3, 4, 10, 11]. At the area level, there have been mixed findings. For example, one study reported no association of hypertension with neighborhood- and city-level education in Argentina [3]. In Brazil, income inequality at the level of Federation Units was not associated with hypertension [4], but another study found that the prevalence of hypertension was significantly higher among residents living in census tracts with lower levels of education [12]. Brazilian Federation Units with a higher Human Development Index had a higher prevalence of hypertension [4]. In Colombia, residents of departments with high-income inequality had a higher prevalence of hypertension [5].

Both individual-level and area-level SES may influence the development of hypertension through a multiplicity of processes. Individual-level SES may be related to hypertension through its impact on behaviors such as diet and physical activity, as well as through stress-related processes [6, 13]. Area-level SES may be a proxy for environmental features such as food, physical activity, health care, and transportation environments [14], and has been correlated with social stressors, such as violence, which may also affect hypertension prevalence [15]. In addition to direct effects, area-level SES can reduce or amplify the effect of individual SES on hypertension [7]. Moreover, both individual-level and area-level SES may influence access to screening, early detection, and treatment of hypertension [16–18].

Latin America has a high prevalence of hypertension in the adult population [2, 19] and a high level of urbanization and inequalities [20], with cities and countries presenting different levels of economic and social development, and different stages of the epidemiological transition [21]. As the prevalence of cardiovascular risk factors increases as a country’s economy grows, the effect of socioeconomic status on hypertension may also change. Therefore, the Latin American context presents a unique opportunity to investigate the associations of individual-level and area-level SES with hypertension. Understanding how social conditions at various levels relate to non-communicable diseases risk factors like hypertension is critical to achieving sustainable development goals [22].

Using unique data compiled and harmonized by the SALURBAL (Salud Urbana en America Latina/Urban Health in Latin America) project [23], we investigated how individual-level and area-level SES are associated with hypertension in adults from 230 cities in eight Latin American countries. In addition, because prior work has found that the social patterning of non-communicable diseases risk factors may vary substantially by gender [3, 6, 8, 10] and country [7, 24, 25], we also examined the extent to which these associations vary by gender and across countries.

## Methods

### Study design

The SALURBAL project compiled and harmonized health, social, and environmental data for all cities above 100,000 inhabitants in eleven Latin American countries [26]. Briefly, SALURBAL defines cities as a single administrative unit (e.g., municipio) or a combination of adjacent administrative units (e.g., several municipios) that are part of the built-up area of the urban agglomeration as determined from satellite imagery. Each “sub-city” is an administrative unit fully nested within a “city”. The “sub-city” units were identified in each country as the smallest geographic administrative units for which health data was easily available. Approximately half of the cities included only one sub-city unit. This study used survey data from 230 cities (encompassing 673 sub-city units) in 8 countries: Argentina, Brazil, Chile, Colombia, El Salvador, Guatemala, Mexico, and Peru (Survey details are provided in Supplementary Material Table S1).

Of 124,743 survey respondents aged 18 years and who resided in SALURBAL cities, we restricted our analyses to those with exposure information both at the individual and area-level, and those with outcome data. The final sample was 109,184 persons from 230 cities (673 sub-cities). Details on exclusions are shown in Figure S1. The median number of individuals per sub-city and city were 65 and 343.5, respectively.

### SALURBAL survey data and outcome

SALURBAL compiled, harmonized, and geocoded individual-level data from nationally representative cross-sectional surveys. For this analysis, we included surveys with hypertension data: Argentina (2013), Brazil (2013), Chile (2010), Colombia (2007), El Salvador (2014), Guatemala (2002), Mexico (2012), and Peru (2016). Surveys were generally conducted by government agencies in different countries for purposes of risk factor surveillance often using similar questions.

The outcome was hypertension. Participants were defined as having hypertension if they reported that a physician had told them that they had hypertension and if they reported using medications “to lower blood pressure” or to control hypertension prescribed by a health care provider (i.e., both conditions had to be fulfilled). We have included the use of drugs in the definition to increase specificity. Gestational hypertension was excluded except in Argentina and Guatemala where the survey questions used did not exclude physician-diagnosed hypertension during pregnancy. This definition was used to incorporate data from as many countries as possible while maximizing comparability across countries.

### Exposures

The three key exposures investigated were individual-level education, a proxy of individual-level SES, and summary scores of sub-city and city education, used as indicators of area-level SES. The sub-city level was included to capture heterogeneity within cities.

Individual-level education was harmonized across countries/surveys and classified into: (1) less than primary (2) primary: individuals who completed primary education, but with incomplete secondary education; (3) secondary: individuals with complete secondary education, complete non-university postsecondary education (e.g., technical school), or with incomplete university education; (4) university or higher: individuals who completed a university degree or with complete/incomplete graduate studies.

The population educational attainment score refers to educational achievement in the overall population. This measure was created using aggregated census data from the individual level of education: (1) the percentage of the population aged 25 years or older that has completed high school level or above, and (2) the percentage of the population aged 25 years or older that has completed university level or above [27]. A score was created by summing the standardized Z-scores of the two variables. Z scores were created for cities, based on the distributions of all cities, and sub-cities, based on the distributions of all sub-cities. Higher score values signify better educational achievement in the population [27].

Other variables included individual-level age (in years) and gender.

### Statistical analysis

All analyses were stratified by gender because of previous evidence of gender differences in the associations between SES and hypertension [3, 6, 8]. For analytical purposes, El Salvador and Guatemala were grouped into one analytical unit named Central America. Descriptive statistics, using frequency distributions, means, and standard deviations (SD), are presented by countries and hypertension status.

The associations of individual-level and area-level SES with hypertension were examined using three-level multilevel logistic regression, including a random intercept for city and sub-city. All models were controlled for age. Model 1 included individual- and city-level education and country as fixed effects. Model 2 included individual- and sub-city-level education and country as fixed effects. Model 3 included all the exposures jointly and country as fixed effects. In Model 4, we included multiplicative interactions between city-level education and country by including the corresponding interaction terms in Model 3. To further explore whether there was heterogeneity in the associations by country, we conducted stratified analyses by country and gender (Supplementary Material Figure S2). Since Peru was the only country that presented a statistically significant and inverse association between city-level education and hypertension, we report associations with city level education separately for two groups (group 1: Argentina, Central America, Brazil, Chile, Colombia, and Mexico; group 2: Peru).

To determine if there were interaction between individual- and area-level SES, we estimated a fifth model (Models 5A and 5B) by including in Model 3 an interaction term between individual- and city-level education (Model 5A) and individual- and sub-city-level education (Model 5B). We plotted the predicted probabilities resulting from the models by individual education category and city- and sub-city-level education.

In order to examine the robustness of our results to using measured blood pressure in the subset of the sample where measures were available (*n* = 50,209) we fitted model 4 using the objective measure to define the outcome: hypertension was defined as systolic blood pressure ≥ 140 mmHg or diastolic blood pressure ≥ 90 mmHg or the use of antihypertensive drugs. These analyses excluded Argentina because blood pressure measurements were not available and were restricted to smaller subsamples in other countries because measurements were not available for all respondents.

The analyses were performed using Stata version 12.0 (StataCorp LP, College Station, USA) and R software. The level of significance was set at 5%. The SALURBAL study protocol was approved by the Drexel University Institutional Review Board with ID #1612005035.

## Results

The individual-level and area-level characteristics of the 109,184 individuals distributed in 673 sub-cities nested in 230 cities, according to country, are presented in Table 1. The median number of participants per sub-city was 65 (interquartile ranges [IQR] = 123) and per city was 343.5 (IQR = 506). The highest percentage of survey participants lived in Brazil and Mexico (24.2% and 23.8%, respectively), while Chile and Central America had the lowest (2.4% and 2.6%, respectively). The overall proportion of hypertension was 13.0% (95%CI = 12.9–13.1). Argentina and Brazil had the highest proportions of hypertension (17.5% and 16.5%, respectively), while Peru (6.5%) and Colombia (7.7%) had the lowest. The mean age of the population studied was 42.7 years (SD = 16.4) and 57.8% were women.

**Table 1:** Individual-level and area-level characteristics of the analytic sample by country. SALURBAL study (*N* = 109,184)

	**TOTAL**	**Argentina**	**Brazil**	**Chile**	**Colombia**	**Mexico**	**Peru**	**Central America **^**a**^
**Sample characteristics**								
**Number of participants**	109,184	21,286	26,398	2,669	18,142	25,995	11,880	2,814
**Number of cities**	230	33	27	19	33	91	23	4
Participants per city								
Median (1stQ-3rdQ)	343.5 (135;641)	509 (415;679)	820 (735;1,100)	84 (33;174)	402 (195;630)	188 (89;387)	311 (162;622)	667.5 (250;1,157)
Min – Max	17 - 3,901	23 - 3,901	513 - 2,543	17 - 793	61 - 3,376	17 - 2,370	90 - 3,278	160 - 1,319
**Number of sub-cities**	673	108	27	70	57	245	149	17
Participants per sub-city								
Median (1stQ-3rdQ)	65 (30;153)	85 (49;338.5)	820 (735;1,100)	20.5 (14;47)	195 (66;402)	62 (33;121)	49 (20;107)	77 (38;160)
Min – Max	5 - 3,092	7 - 885	513 - 2,543	5 - 239	14 - 3,092	14 - 835	5 - 521	18 - 1,319
**Area-level characteristics**								
City-level education, median (1stQ-3rdQ)	0.13 (-0.66;1.02)	-0.54 (-1.12;-0.33)	0.76 (0.25;1.16)	-1.06 (-1.42;0.69)	-1.17 (-0.86;0.32)	0.25 (-0.95;0.52)	2.93 (1.33;3.49)	-1.71 (-1.71;-0.84)
Sub-city-level education, median (1stQ-3rdQ)	0.25 (-0.56;1.19)	-0.34 (-0.74;-0.07)	1.19 (0.60;1.72)	- 0.65 (-1.08;-0.22)	0.07 (-0.48-0.64)	-0.14 (-0.98;0.57)	1.20 (0.58;2.14)	-1.65 (-1.65;-1.39)
**Individual-level characteristics**								
Age, mean (SD)	42.7 (16.4)	44.8 (17.9)	43.4 (16.6)	46.8 (17.6)	39.1 (14.0)^b^	43.6 (16.1)	39.7 (15.6)	42.9 (16.6)
Women, n (%)	63,126 (57.8)	11,991 (56.3)	15,692 (59.4)	1,601 (60.0)	10,366 (57.1)	14,760 (56.8)	6,835 (57.5)	1,881 (66.8)
Educational level, n (%)								
Less than primary	18,856 (17.3)	1,982 (9.3)	5,626 (21.3)	284 (10.6)	3,104 (17.1)	5,361 (20.6)	1,516 (12.8)	983 (34.9)
Primary	36,674 (33.6)	7,841 (36.8)	5,724 (21.7)	922 (34.5)	6,269 (34.6)	12,674 (48.8)	2,065 (17.4)	1,179 (41.9)
Secondary	39,310 (36.0)	7,865 (36.9)	10,214 (38.7)	1,239 (46.3)	7,150 (39.4)	5,439 (20.9)	6,845 (57.6)	561 (19.9)
University or higher	14,344 (13.1)	3,598 (16.9)	4,834 (18.3)	227 (8.5)	1,619 (8.9)	2,521 (9.7)	1,454 (12.2)	91 (3.2)
Hypertension, n (%)	14,208 (13.0)	3,725 (17.5)	4,364 (16.5)	358 (13.4)	1,399 (7.7)	3,158 (12.1)	773 (6.5)	431 (15.3)
Women	9,355 (14.8)	2,317 (19.3)	2,891 (18.4)	259 (16.2)	961 (9.3)	2,111 (14.3)	480 (7.0)	336 (17.9)
Men	4,853 (10.5)	1,408 (15.5)	1,473 (13.8)	99 (9.3)	438 (5.6)	1,047 (9.3)	293 (5.8)	95 (10.1)

^a^Central America: El Salvador and Guatemala grouped together

^b^Residents of Colombia had a lower average age, as the survey in this country did not include individuals over 70 years of age

The proportion of self-reported hypertension was 10.5% (95%CI = 10.2–10.8) for men and 14.8% (95%CI = 14.5–15.1) for women (Table 2). Participants with hypertension were, on average, older and concentrated in the lower education categories as compared with those without hypertension, both for men and women. Also, individuals with hypertension had slightly higher sub-city-level education and lower city-level education than those without hypertension for both sexes.

**Table 2:** Individual-level and area-level characteristics of the analytical sample by hypertension status and gender

	**Gender**					
	**Women**			**Men**		
	**No hypertension**	**Hypertension**	***p*-value**^**a**^	**No hypertension**	**Hypertension**	***p*-value**^**a**^
	***N* = 53,771 (85.2%)**	***N* = 9,355 (14.8%)**		***N* = 41,205 (89.5%)**	***N* = 4,853 (10.5%)**	
**Individual characteristics**						
Age, mean (SD)	39.8 (15.0)	60.9 (13.6)	< 0.001	40.2 (15.2)	59.9 (13.5)	< 0.001
Educational level, n (%)			< 0.001			< 0.001
Less than primary	8,170 (15.2)	3,396 (36.3)		6,092 (14.8)	1,198 (24.7)	
Primary	17,835 (33.2)	3,111 (33.2)		14,112 (34.2)	1,616 (33.3)	
Secondary	20,423 (38.0)	1,904 (20.3)		15,694 (38.1)	1,289 (26.6)	
University or higher	7,343 (13.7)	944 (10.1)		5,307 (12.9)	750 (15.45)	
**Contextual characteristics**						
Sub-city-level education, mean (SD)	0.31 (1.27)	0.34 (1.27)	< 0.001	0.29 (1.26)	0.38 (1.29)	< 0.001
City-level education, mean (SD)	0.26 (1.43)	0.11 (1.28)	< 0.001	0.26 (1.43)	0.15 (1.29)	< 0.001

^a^*p*-values refer to Kruskal–Wallis tests and chi-square tests (for categorical variables). Comparing hypertension yes/no

Table 3 shows associations of individual-, sub-city- and city-level education with hypertension. The random intercepts remained significant for all models in both sexes, indicating residual variability in hypertension between cities and sub-cities. In women, higher individual-level education was associated with lower odds of hypertension in all models. In contrast, higher sub-city-level education was associated with higher odds of hypertension in all models. City-level education was not associated with hypertension even after joint adjustment of the three exposure variables, age, and country-fixed effects (Model 3). However, model 4 reveled heterogeneity in the associations of city education with hypertension by country: higher city-level education was associated with lower odds of hypertension in Peru but no association was observed for Argentina, Brazil, Central America, Chile, Colombia, and Mexico.

**Table 3:** Associations of individual-, city- and sub-city-level education, with hypertension in 230 cities in Latin America

**Individual, city, and sub-city characteristics**^**a**^	**Model 1**	**Model 2**	**Model 3**	**Model 4**
	**OR (95%CI)**	**OR (95%CI)**	**OR (95%CI)**	**OR (95%CI)**
**Women (*N* = 63,126)**				
*Individual-level education*				
Less than primary	Ref	Ref	Ref	Ref
Primary	0.99 (0.93-1.06)	0.99 (0.92-1.06)	0.99 (0.92-1.06)	0.98 (0.92-1.05)
Secondary	**0.76 (0.70**-**0.82)**	**0.75 (0.70**-**0.81)**	**0.75 (0.70**-**0.81)**	**0.76 (0.70**-**0.82)**
University or higher	**0.67 (0.61**-**0.74)**	**0.66 (0.60**-**0.73)**	**0.66 (0.60**-**0.73)**	**0.67 (0.61**-**0.74)**
*City-level education*	0.98 (0.93-1.04)		0.95 (0.89-1.01)	
*Sub-city-level education*		**1.04 (1.01**-**1.08)**	**1.05 (1.01**-**1.10)**	**1.07 (1.02**-**1.12)**
Argentina, Central America, Brazil, Chile, Colombia, and Mexico				
City-level education	**-**	**-**	**-**	1.01 (0.94-1.08)
Peru				
City-level education				**0.79 (0.64**-**0.97)**
Intercept variance (SE) (city)	**0.0362 (0.1904)**	**0.0373 (0.1931)**	**0.0354 (0.1884)**	**0.0641 (0.2533)**
Intercept variance (SE) (sub-city)	**0.0262 (0.1622)**	**0.0241 (0.1555)**	**0.0258 (0.1608)**	**0.0272 (0.1652)**
**Men (*N* = 46,058)**				
*Individual-level education*				
Less than primary	Ref	Ref	Ref	Ref
Primary	**1.51 (1.37**-**1.66)**	**1.49 (1.36**-**1.64)**	**1.50 (1.36**-**1.65)**	**1.48 (1.35**-**1.63)**
Secondary	**1.59 (1.43**-**1.76)**	**1.56 (1.40**-**1.73)**	**1.56 (1.40**-**1.73)**	**1.58 (1.42**-**1.75)**
University or higher	**1.68 (1.49**-**1.88)**	**1.63 (1.45**-**1.83)**	**1.63 (1.45**-**1.83)**	**1.65 (1.47**-**1.86)**
*City-level education*	0.98 (0.92-1.04)		**0.92 (0.85**-**0.99)**	
*Sub-city-level education*		**1.06 (1.01**-**1.12)**	**1.09 (1.03**-**1.16)**	**1.11 (1.05**-**1.18)**
Argentina, Central America, Brazil, Chile, Colombia, and Mexico				
City-level education	**-**	**-**	**-**	1.00 (0.92-1.10)
Peru				
City-level education	**-**	**-**	**-**	**0.77 (0.59**-**0.99)**
Intercept variance (SE) (city)	**0.0221 (0.1487)**	**0.0270 (0.1644)**	**0.0213 (0.1460)**	**0.1001 (0.3164)**
Intercept variance (SE) (sub-city)	**0.0233 (0.1528)**	**0.0154 (0.1244)**	**0.0191 (0.1384)**	**0.0250 (0.1582)**

Combinations of the main effect of city-level education and the interaction coefficient were used to derive estimates for different countries

Model 1: Individual- and city-level education, adjusted for country-fixed effect. Model 2: Individual- and sub-city-level education, adjusted for country-fixed effect. Model 3: Individual-, sub-city- and city-level education, adjusted for country-fixed effect. Model 4: Model 3 and an interaction term between city-level education and different country groups (*p*-value for interaction in women = 0.004 and men = 0.012)

Bold values have a *p*-value < 0.05

*Ref.* Reference group, *OR* Odds Ratio, *95%CI* 95% confidence intervals, *SE* Standard error

^a^All models were adjusted for individual age. City- and sub-city-level education (educational attainment score) was standardized to a mean of 0 and a standard deviation (SD) of 1. The OR is estimated for a 1 SD (0.30847) and (0.24469) difference in sub-city- and city-educational attainment scores, respectively

In men, higher individual- and sub-city-level education were associated with higher odds of hypertension in all models (Table 3). In contrast, higher city-level education was associated with lower odds of hypertension in the model with joint adjustment of the three exposure variables, age, and country-fixed effects. Exploration of interactions showed that this association was stronger in Peru while no association was observed for Argentina, Brazil, Central America, Chile, Colombia, and Mexico.

**Fig. 1:**
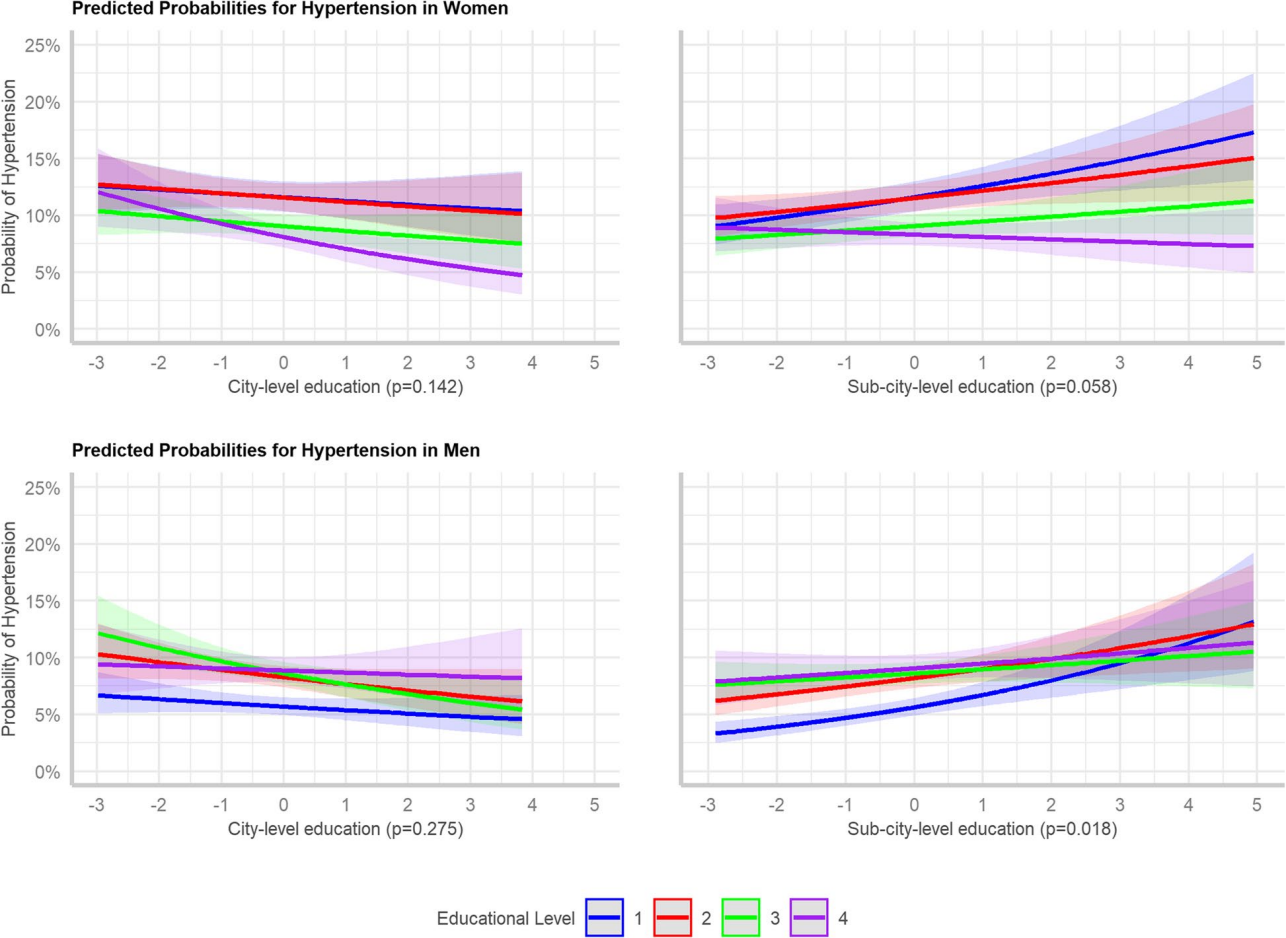
Predicted probabilities of hypertension based on the regression models to assess the effect modification of individual-level education by area-level education. The *p*-value presented refers to the global interaction test. Education level: 1 = less than primary; 2 = primary; 3 = secondary; 4 = university or higher. Sub-city- and city-educational attainment score values are based on the standardized variable used in the model so the range of values differs from the one presented in Table 1

Figure 1 shows adjusted predicted probabilities of hypertension for individual education by levels of the city- and sub-city education. Although interaction terms were not always statistically significant, interesting patterns were observed. In women, the inverse association of individual-level education with hypertension became stronger at higher levels of city and sub-city education. In men, inverse associations of hypertension with education appeared to emerge at high levels of sub-city education. Estimates from models with interactions are shown in a supplementary table (Supplementary Material Table S2).

In sensitivity analyses, restricted to persons with measured blood pressure or using antihypertensive drugs, we observed similar results in women in terms of directionality and patterns of associations for all three exposures in the full model (Supplementary Material Table S3). However, in men in contrast to what was observed with self-reported hypertension, the highest individual-level education category was associated with lower odds of hypertension (OR *versus* less than primary: 1.14 [95%CI = 1.02–1.27], 1.01 [95%CI = 0.91–1.12], 0.86 [95%CI = 0.76–0.97], for primary, secondary and university or higher categories respectively). Findings for the city- and sub-city-level education were similar in directionality to those reported in the main analysis.

## Discussion

We investigated associations of individual-level and area-level education with hypertension in adults from 230 Latin American cities. We found a clear gradient across individual-level education, but in opposite direction between genders. In women, higher education levels were associated with a lower proportion of hypertension. In men, higher education levels were associated with a higher proportion of hypertension. Although interaction terms between individual-level and area-level education were not always statistically significant, descriptive analyses suggested that an inverse association of individual-level education with hypertension became stronger or emerged as city or sub-city education increased.

Our results also demonstrated that higher sub-city-level education was associated with higher odds of hypertension in both genders. Moreover, the association of city-level education with hypertension varied across countries. In Peru, there was an inverse association (higher city education was associated with lower proportion of hypertension), while in other countries there was no association in women or men.

A recent meta-analysis of 51 studies found that educational attainment was a stronger predictor of hypertension prevalence than income or occupation [6]. However, to our knowledge, transnational studies investigating associations of individual-level and area-level SES with hypertension using the same indicator at different levels and in different countries have not been conducted.

We found that self-reported hypertension was inversely associated with education among women, but positively associated with education among men. Evidence limited from LMICs generally shows a higher prevalence of hypertension in women with lower education levels compared with higher education, while for men, evidence is mixed [3, 11, 28, 29]. Explanations for gender disparities may include more physically demanding jobs for less educated men [30] or differential patterning of other risk factors by SES in women and men [31]. Moreover, being a woman and having low education may be linked to higher exposure to chronic stress conditions, such as informal employment, single parenthood and role overload, violence, and stress at home [32, 33].

Furthermore, women’s appearance is heavily emphasized in patriarchal societies [34], with heteronormative gender norms often shaping more educated women’s behaviors [35] (e.g. they are more likely to face pressure to adjust their bodies to social expectations) [35]. Surveillance bias may also explain part of the association observed between education and hypertension in men. Men often search less for health systems and medical advice [18] and this could be especially pronounced in lower SES men which could explain the strong positive association of individual-level education with self-reported hypertension that we observed.

Our sensitivity analysis based on objective measures of blood pressure showed a different pattern for individual level education in men: the highest education category had significantly lower odds than the lowest category. This is consistent with the argument that differences in access and utilization of health care by SES in men could explain the positive association of individual-level SES with hypertension that we observed. Of note the gender differences in associations of education with hypertension that we report here as similar to those reported by Braverman et al. [24] for diabetes and Mazariego et al. [25] for obesity in SALURBAL in previous work.

Our study also showed sub-city and city contextual effects. After accounting for individual education, we found a positive association between sub-city-level education and hypertension for both genders. Lower access to health care (and consequent diagnosis of hypertension) in areas of lower education could at least partly explain this finding [16–18]. However, we observed a similar pattern when objective measures of hypertension were used. In consonance with our findings, a positive association between area-level SES and hypertension was also previously reported in Brazil using objective hypertension measures [4].

The mechanisms underlying a positive association of sub-city education with hypertension may include other factors associated with area-level SES including the nature of work [36], access to and consumption of processed foods [37], sedentary behaviors [38, 39], promoted by work and urban environments (e.g., car dependence), or even factors such as levels of pollution, heat, and noise, all of which have been linked to hypertension [40].

Significant associations between city-level education and hypertension were limited to individuals residing in Peru; higher educational attainment of the population at the city level was associated with a lower proportion of hypertension in women and men. While the highest global prevalence of hypertension was observed in some Latin American and Caribbean countries, the lowest global prevalence of hypertension was found in Peru [2, 19] and the stages of the hypertension of the epidemic could be linked to social patterning and differences across cities. Country differences in access to care and the patterning of access to care by city SES could also play a role when self-reported hypertension is used as a hypertension indicator. The positive association between city-level education and hypertension in Peru is a question that deserves additional research.

Our results also suggest possible interactions between contextual- and individual-level education. In women, the inverse association of individual-level education with hypertension became stronger as sub-city and city education increased. In men, the positive association of individual-level education with hypertension was lost, and an inverse gradient emerged (higher education, lower hypertension prevalence) as sub-city education increased. This is consistent with findings from prior works showing that inverse social gradients in cardiovascular risks emerge as contextual education increases [24, 25]. It may be related to the social patterning of risk factors for hypertension that emerges as socioeconomic development increases.

This study has some limitations. First, we use a cross-sectional design, which does not allow us to draw causal inferences; however, descriptive information is also important to public policy. Second, the ascertainment of hypertension status was through self-report, which may have led to differential information bias, with groups with less access to healthcare under-represented, and consequently, resulting in underestimates of inverse education gradients if lower SES groups have lower access to care. Third, gestational hypertension was not excluded in Argentina and Guatemala. Gestational hypertension data will be limited to some women who were pregnant at the time of the survey. In countries where we have information on current pregnancy (Brazil, Chile, Mexico, Peru and El Salvador), the presence of this condition was reported by approximately 4% of women aged 18 to 49 years, with only 19 (1.8%) of these were considered hypertensive. Therefore, we do not believe this is likely to have had a significant impact on our results. Fourth, we did not adjust for hypertension risk factors such as physical activity, diet, smoking, obesity, and diabetes because we view them as likely mediators of the associations we are investigating [15, 41]. In addition, it is not possible to rule out residual confounding due to unmeasured or unknown factors. Fifth, survey years are not always aligned with the census years from which area-level education information was drawn. Finally, despite efforts to harmonize surveys across countries, some heterogeneity may still exist and affect our results. Nonetheless, to attenuate these potential remaining differences we used the country as fixed effects for the main analyses.

On the other hand, this study has several strengths. To our knowledge, this is the first transnational study to examine the association between individual- and area-level SES with hypertension using the same indicator at different levels. Second, our study included a large sample of individuals (109,184) and cities (230) representing a significant proportion of the urban population of Latin America and used a large harmonized dataset. Third, our multilevel approach allowed us to analyze individual and macro-level contextual factors. In addition, the associations were adjusted for country-fixed effects removing the effect for unmeasured country factors such as differences in healthcare and education systems across countries.

In conclusion, our results demonstrate gender and social inequalities in hypertension in Latin American cities. First, we identified gender differences in the relationship between individual education and hypertension, with higher individual-level education associated with lower odds of hypertension among women and higher odds among men. Second, we identified that higher sub-city-level education was positively associated with hypertension in both women and men. Third, we identified that higher city-level education was associated with lower odds of hypertension in both sexes in Peru. Thus, our results suggest that strategies to deal with the burden of hypertension in LIMCs should adopt equity-based and context-sensitive efforts.

### Supplementary Information

**Additional file 1: Figure S1.** Flowchart of the process used to define the sample used in the paper. **Table S1.** Characteristics of the national surveys included in our sample. **Figure S2.** Associations of individual-, city- and sub-city-level education, with hypertension country-stratified. SALURBAL study (*N* = 109,184). **Table S2.** Associations between Individual-, city- and sub-city-level education with hypertension for models containing an interaction term between individual-level education with city- and sub-city education. **Table S3.** Sensitivity analyses: associations between individual-, sub-city- and city-level education with hypertension (self-reported vs. objectively measured).

## Data Availability

Data are available upon reasonable request. The SALURBAL study obtained data from health and/or statistical agencies within each country. Some data sources are available under restricted access due to data use agreements between the SALURBAL Study and statistical agencies within the country. Requests for the harmonized data can be obtained by contacting the SALURBAL Data Methods Core (salurbal.data@drexel.edu) and after completing data use agreements. To learn more about SALURBAL’s dataset, visit https://drexel.edu/lac/ or contact the project at salurbal@drexel.edu.

## Abbreviations

OR: Odds Ratio
CI: Confidence interval
SD: Standard deviation
IQR: Interquartile ranges
SALURBAL: Salud Urbana en America Latina/Urban Health in Latin America
LMICs: Low- and middle-income countries
SES: Socioeconomic status
