# Supplementary material for: Gender differences in the association of individual and contextual socioeconomic status with hypertension in 230 Latin American cities from the SALURBAL study: a multilevel analysis
Source: BMC Public Health. 2023 Aug 11;23:1532. doi: 10.1186/s12889-023-16480-3 (PMC10416382; doi:10.1186/s12889-023-16480-3)
Supplement: Supplementary file 1 — Additional file 1: Figure S1. Flowchart of the process used to define the sample used in the paper. Table S1. Characteristics of the national surveys included in our sample. Figure S2. Associations of individual-, city- and sub-city-level education, with hypertension country-stratified. SALURBAL study (N = 109,184). Table S2. Associations between Individual-, city- and sub-city-level education with hypertension for models containing an interaction term between individual-level education with city- and sub-city education. Table S3. Sensitivity analyses: associations between individual-, sub-city- and city-level education with hypertension (self-reported vs. objectively measured). [file 12889_2023_16480_MOESM1_ESM.pdf]

SUPPLEMENTAL MATERIAL

Gender differences in the association of individual and contextual socioeconomic status with hypertension in 230 Latin American cities from the SALURBAL study: a multilevel analysis

Multilevel logistic model specification

The "fully adjusted" model (model 3) in the paper corresponds to

$$Y_{ijk}|X_{ijk} \sim \text{Bernoulli}(\pi_{ijk}),$$

where Y_{ijk} is the indicator of hypertension (= 1 if the individual reported hypertension and 0 otherwise) reported by the ith individual nested within the jth subcity nested within the kth city, $i = 1, \dots, I, j = 1, \dots, J, k = 1, \dots, K$ (there are a total of I = 109,184 individuals, J = 673 sub-cities and K = 230 cities in the sample). Here, X_{ijk} represents all the conditional information in the model (i.e. exposures, random effects and other covariates). Since a logit link was assumed, the logit of the conditional probability of reporting hypertension π_{ijk} satisfies

$$\text{logit}(\pi_{ijk}) = \beta_0 + \beta_1 A_{ijk} + \alpha_i + \alpha_j + \sum_{m=1}^{6} \gamma_m C_k^m + \sum_{l=1}^{3} \tau_l IE_{ijk}^l + \theta SE_{jk} + \eta CE_k,$$

where β_0 is the overall intercept, A_{ijk} is individual age (in years), α_i is a sub-city-level random intercept, α_j is a city-level random intercept, the C_k^m's are country-level indicators (reference = Argentina), the IE_{ijk}^l's are individual education indicators (reference = less than complete primary), SE_{jk} is the sub-city-level education attainment score, CE_k is the city-level education attainment score and $\beta_1, \gamma_1, \gamma_2, \gamma_3, \gamma_4, \gamma_5, \gamma_6, \tau_1, \tau_2, \tau_3, \theta, \eta$ are regression coefficients. All models are stratified by sex.

Country-stratified analyses

The models for the country-stratified analyses are all particular instances of the fully adjusted model defined above, and are obtained by removing the country-level fixed effects $\gamma_m C_k^m$'s from the model and restricting the sample to each specific country separately.

Interaction analyses

For the analyses of interactions, we assume slightly more general versions of the fully adjusted model (model 3) used for the main results.

1. *City-level education and countries* (model 4). Countries were classified into 2 groups (group 1 – Argentina, Brazil, Central America, Chile, Colombia, Mexico; group 2 – Peru), and thus the term $\sum_{m=1}^{6} \gamma_m C_k^m$ is replaced with a single country-level fixed effect $\gamma_1 \bar{C}_k$, with group 1 as the reference. In order to capture the interaction effect between city-level education and country, the term $\delta CE_k \bar{C}_k$ was introduced in the model.

2. *City-level education and individual education* (model 5A). In order to capture the interaction effect, the terms $\delta_1 CE_k IE^1_{ijk}, \delta_2 CE_k IE^2_{ijk}, \delta_3 CE_k IE^3_{ijk}$ (that is, one for each individual education category) were introduced in the model.
3. *Sub-city-level education and individual education* (model 5B). In order to capture the interaction effect, the terms $\delta_1 SE_{jk} IE^1_{ijk}, \delta_2 SE_{jk} IE^2_{ijk}, \delta_3 SE_{jk} IE^3_{ijk}$ (that is, one for each individual education category) were introduced in the model.

List of Figures and Tables

Figure S1 shows a flowchart of the process used to define the sample used in the paper, and, Figure S2 shows a forest plot of the country-stratified associations between arterial hypertension and the exposures estimated in the fully adjusted model (model 3).

Table S1 shows a characteristics of the national surveys included in our sample, Table S2 contains estimated associations between arterial hypertension and the exposures for models 5A and 5B, and Table S3 contains estimated associations between arterial hypertension (self-reported vs. objectively measured) and the exposures for the model with an interaction between city-level education and country groups (model 4).

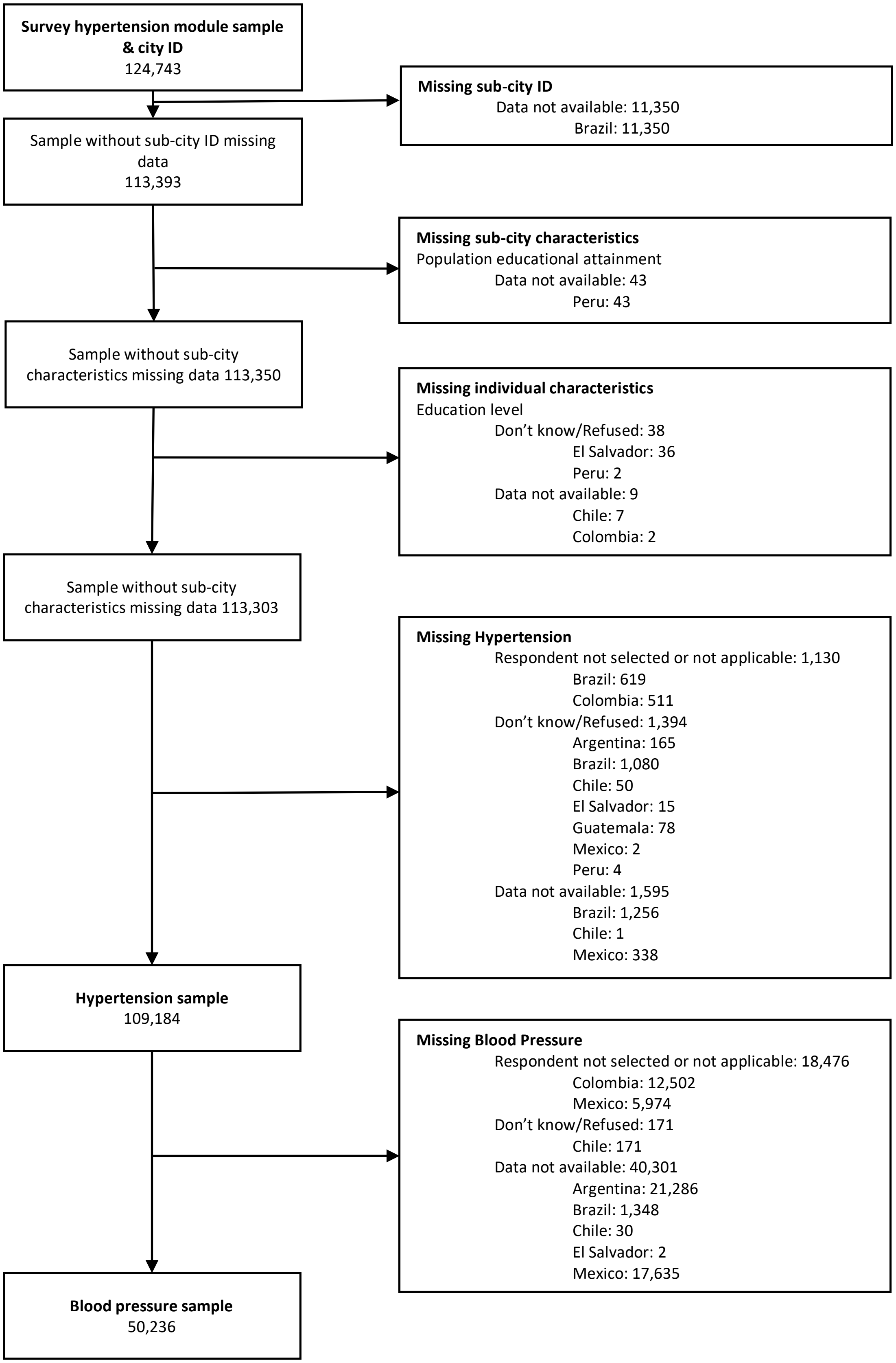

Figure S1. **Flowchart of the process used to define the sample used in the paper.**

Table S1: Characteristics of the national surveys included in our sample.

Country	Survey	Sample Characteristics	Sampling Strategy
Argentina	Survey: Encuesta Nacional de Factores de Riesgo, ENFR (National Risk Factors Survey)<br>Year: 2013	Age: >18 years<br>Total N: 32,365<br>N in SALURBAL: 21,451<br>N selected: 21,451<br>N used: 21,286	Multistage [Aglomerado censal; área (groups of radio censales); household; person 18 years or older]<br>Stratified [population size; education level of head of household]
Brazil	Survey: Pesquisa Nacional de Saúde, PNS (National Health Survey)<br>Year: 2013	Age: All ages<br>Total N: 64,308 adults 18+ years<br>N in SALURBAL: 29,353<br>N selected: 29,353<br>N used: 26,398	Multistage: census tracts or groups of census tracts; households; person 18 years or older<br>Stratified: capital city, metropolitan region, or integrated economic development region, then rest of municipalities; Urban/rural; total household income
Chile	Survey: Encuesta Nacional de Salud, ENS (National Health Survey)<br>Year: 2010	Age: ≥15 years<br>Total N: 5,293<br>N in SALURBAL: 2,840<br>N selected: 2,727 adults 18+ years<br>N used: 2,669	Multistage: Comunas; Segments within comunas; household; person 15 years or older<br>Stratified: urban/rural with three groups of population sizes
Colombia	Survey: Encuesta Nacional de Salud, ENS (National Health Survey)<br>Year: 2007	Age: 0 - 69 years<br>Total N: 102,677 (43,182 adults 18-69 years)<br>N in SALURBAL: 43,182 (18,824 adults 18-69 years competed module 2 or module 4)<br>N selected: 18,654<br>N used: 18,143	Multistage: Municipalities or combination of municipalities if small, Manzanas; household; person adults 18-69 and all children 17 and under<br>Stratified: region; urbanization of municipal seats; urban/rural municipal population; unsatisfied basic needs
Mexico	Survey: Encuesta Nacional de Salud y Nutricion, ENSANUT (National Survey for Health and Nutrition)<br>Years: 2012	Age: all ages<br>Total N: 46,277 adults 18+ years<br>N in SALURBAL: 26,335 adults<br>N selected: 26,335<br>N used: 25,995	Multistage: AGEB; Manzana (urban) or pseudo-manzanas withig localidades (rural); Households; 1 person within each of the groups (0-4 years, 5-9 years, 10-19 years, 20 years and older, recent medical service user)<br>Stratified: socioeconomic status of AGEB at the state level
Peru	Survey: Encuesta Nacional de Demografia y Salud, ENDES (National Survey of Demographics and Health)<br>Year: 2016	Age: All ages<br>Total N: 122,368 (32158 adults 18-69 years)<br>N in SALURBAL: 12,597 adults 18+ years<br>N selected: 11,929<br>N used: 11,880	Multistage: Conglomerado (set of census blocks – urban) or Empadronamiento (set of households – rural); Households; One person within each of the groups (>15 years, females 15-49 years, children <5 years, children <12 years)<br>Stratified: Department; Urban/Rural
El Salvador	Survey: Encuesta Nacional de Enfermedades Cronicas no transmisibles en Poblacion Adulta de El Salvador ENECA (National Survey of Noncommunicable Chronic Diseases in the Adult Population of El Salvador)<br>Year: 2014-2015	Age: ≥20 years<br>Total N: 4,817<br>N in SALURBAL: 1,546<br>N selected: 1,546<br>N used: 1,495	Two-stage [Segmento censal, groups of dwellings (compacto); all household members 20 years and older]
Guatemala	Survey: Encuesta Multi-nacional de Diabetes mellitus y Factores de Riesgo, CAMDI (Multina-tional Survey of Diabetes Mellitus & Risk Factors, Central American Diabetes Initiative)<br>Year: 2002-2003	Age: ≥20 years<br>Total N: 1,397<br>N in SALURBAL: 1,397<br>N selected: 1,397<br>N used: 1,319	Multistage: Segmento censal, groups of dwell-ings (compacto); all household members 20 years and older

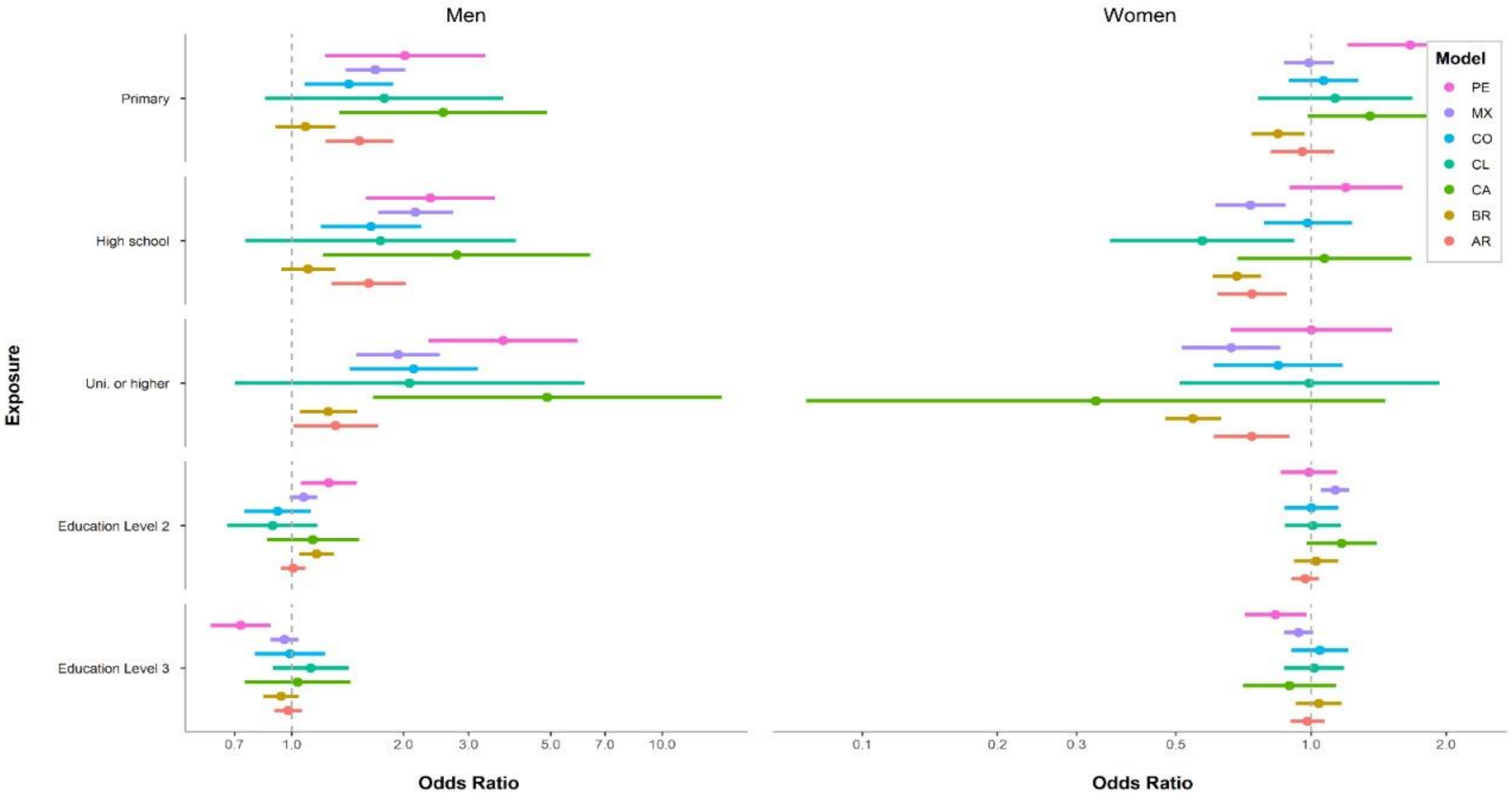

Figure S2. Associations of individual-, city- and sub-city-level education, with hypertension country-stratified. SALURBAL study (N = 109,184).
Multilevel structure: individuals nested within sub-cities, nested within cities. Analyses adjusted by individual age

Table S1. Associations between Individual-, city- and sub-city-level education with hypertension for models containing an interaction term between individual-level education with city- and sub-city education.

**Individual, sub-city and city characteristics (N = 109,184)**	***WOMEN*** OR (95% CI)	***MEN*** OR (95% CI)
**Model 5A**		
City-level education	0.96 (0.89-1.04)	0.94 (0.85-1.03)
Sub-city-level education	**1.06 (1.01-1.10)**	**1.09 (1.03-1.16)**
Individual-level education		
Less than primary	Ref.	Ref.
Primary	0.99 (0.93-1.06)	**1.49 (1.35-1.65)**
Secondary	**0.75 (0.70-0.81)**	**1.56 (1.41-1.73)**
University or higher	**0.67 (0.61-0.73)**	**1.61 (1.43-1.81)**
Individual education * City education		
Less than primary * City education	0.96 (0.89-1.04)	0.94 (0.85-1.03)
Primary * City education	0.96 (0.85-1.08)	0.92 (0.77-1.09)
Secondary * City education	0.95 (0.84-1.07)	0.87 (0.74-1.04)
University or higher * City education	**0.86 (0.75-0.99)**	0.97 (0.81-1.17)
Intercept variance (SE) (city)	**0.0347 (0.1863)**	**0.0214 (0.1466)**
Intercept variance (SE) (sub-city)	**0.0262 (0.1621)**	**0.0187 (0.1370)**
Global p-value for interactions	0.142	0.275
**Model 5B**		
City-level education	0.95 (0.89-1.01)	**0.91 (0.85-0.98)**
Sub-city-level education	**1.09 (1.03-1.16)**	**1.20 (1.10-1.31)**
Individual-level education		
Less than primary	Ref.	Ref.
Primary	0.99 (0.92-1.06)	**1.49 (1.36-1.65)**
Secondary	**0.75 (0.70-0.82)**	**1.58 (1.42-1.75)**
University or higher	**0.69 (0.62-0.76)**	**1.67 (1.48-1.88)**
Individual education* Sub-city education		
Less than primary * Sub-city education	**1.09 (1.03-1.16)**	**1.20 (1.10-1.31)**
Primary * Sub-city education	1.06 (0.95-1.19)	1.10 (0.94-1.30)
Secondary * Sub-city education	1.05 (0.93-1.17)	1.04 (0.88-1.23)
University or higher * Sub-city education	0.97 (0.85-1.10)	1.05 (0.88-1.25)
Intercept variance (SE) (city)	**0.0344 (0.1856)**	**0.0200 (0.1417)**
Intercept variance (SE) (sub-city)	**0.0257 (0.1604)**	**0.0196 (0.1401)**
Global p-value for interactions	0.059	**0.018**

Ref.: reference group. OR: Odds Ratio. 95%CI: 95% confidence intervals. SE: standard error. Bold values have a p-value<0.05.

Multilevel structure: individuals nested within sub-cities, nested within cities. Analyses adjusted by age and country-fixed effect. **Interaction model A:** include an interaction term individual-level education*city-level education. **Interaction model B:** include interaction term individual-level education*sub-city-level education. Combinations of the main effect of individual-level education and the interaction coefficient were used to derive the estimates.

Table S2. Sensitivity analyses: associations between individual-, sub-city- and city-level education with hypertension (self-reported vs. objectively measured). SALURBAL study (N = 50,236).

**Individual, sub-city and city characteristics (N = 50,236)***	***Self-reported***** **OR (95%CI)**	***Measured***** **OR (95%CI)**
**WOMEN (N= 29,806)**		
City-level education		
Sub-city-level education	**1.12 (1.04-1.21)**	**1.08 (1.01-1.15)**
Individual-level education		
Less than primary	REF	**REF**
Primary	1.03 (0.92-1.14)	0.97 (0.88-1.06)
Secondary	**0.77 (0.70-0.86)**	**0.70 (0.64-0.77)**
University or higher	**0.63 (0.55-0.72)**	**0.51 (0.45-0.57)**
Brazil, Central America, Chile, Colombia, Mexico		
City-level education	1.07 (0.95-1.21)	1.01 (0.92-1.11)
Peru		
City-level education	**0.76 (0.58-0.99)**	**0.79 (0.64-0.99)**
Intercept variance (SE) (city)	**0.0374 (0.1935)**	**0.0325 (0.1804)**
Intercept variance (SE) (sub-city)	**0.0635 (0.2520)**	**0.0238 (0.1544)**
Global p-value for interactions	**0.000**	**0.002**
**MEN (N= 20,403)**		
City-level education		
Sub-city-level education	**1.25 (1.14-1.37)**	1.06 (0.99-1.13)
Individual-level education		
Less than primary	REF	REF
Primary	1.22 (1.05-1.42)	**1.14 (1.02-1.27)**
Secondary	**1.31 (1.13-1.52)**	1.01 (0.91-1.12)
University or higher	**1.59 (1.36-1.87)**	**0.86 (0.76-0.97)**
Brazil, Central America, Chile, Colombia, Mexico		
City-level education	1.03 (0.90-1.18)	0.99 (0.89-1.10)
Peru		
City-level education	**0.72 (0.53-0.96)**	0.93 (0.73-1.17)
Intercept variance (SE) (city)	**0.0344 (0.1855)**	**0.0472 (0.2174)**
Intercept variance (SE) (sub-city)	**0.0191 (0.1384)**	**0.0220 (0.1483)**
Global p-value for interactions	**0.000**	**0.436**

Ref.: reference group. OR: Odds Ratio. 95%CI: 95% confidence intervals. SE: standard error. Bold values have a p-value<0.05.

* These analyses excluded Argentina because blood pressure measurements were not available and were restricted to smaller subsamples in other countries because measurements were not available for all respondents.

** Participants were defined as having hypertension if they reported that a physician told them that they had hypertension and if they reported using medications to lower blood pressure prescribed by a healthcare provider (i.e., both conditions had to be fulfilled).

*** Hypertension was defined as systolic blood pressure ≥ 140 mmHg or diastolic blood pressure ≥ 90 mmHg or use of antihypertensive drugs.

Models: Individual-, sub-city- and city-level education, an interaction term between city-level education and different country groups, and fixed control for age. Combinations of the main effect of city-level education and the interaction coefficient were used to derive estimates for different countries.
